# Non-canonical PD-1 signaling in cancer and its potential implications in clinic

**DOI:** 10.1136/jitc-2020-001230

**Published:** 2021-02-16

**Authors:** Haoran Zha, Ying Jiang, Xi Wang, Jin Shang, Ning Wang, Lei Yu, Wei Zhao, Zhihua Li, Juan An, Xiaochun Zhang, Huoming Chen, Bo Zhu, Zhaoxia Li

**Affiliations:** 1 Department of Oncology, PLA Rocket Force Characteristic Medical Center, Beijing, P.R. China; 2 Postgraduate Training Base in Rocket Army Special Medical Center of the PLA, Jinzhou Medical University, Jinzhou, P.R. China; 3 Otorhinolaryngology, PLA Rocket Force Characteristic Medical Center, Beijing, P.R. China; 4 Department of Health Service, Guard Bureau of the Joint Staff Department, Central Military Commission of PLA, Beijing, P.R. China; 5 Institute of Cancer, Xinqiao Hospital, Third Military Medical University, Chongqing, P.R. China

**Keywords:** programmed cell death 1 receptor, tumor microenvironment, biomarkers, tumor, immunotherapy

## Abstract

Programmed cell death 1 (PD-1)-based immunotherapy has revolutionized the treatment of various cancers. However, only a certain group of patients benefit from PD-1 blockade therapy and many patients succumb to hyperprogressive disease. Although, CD8 T cells and conventional T cells are generally considered to be the primary source of PD-1 in cancer, accumulating evidence suggests that other distinct cell types, including B cells, regulatory T cells, natural killer cells, dendritic cells, tumor-associated macrophages and cancer cells, also express PD-1. Hence, the response of patients with cancer to PD-1 blockade therapy is a cumulative effect of anti-PD-1 antibodies acting on a myriad of cell types. Although, the contribution of CD8 T cells to PD-1 blockade therapy has been well-established, recent studies also suggest the involvement of non-canonical PD-1 signaling in blockade therapy. This review discusses the role of non-canonical PD-1 signaling in distinct cell types and explores how the available knowledge can improve PD-1 blockade immunotherapy, particularly in identifying novel biomarkers and combination treatment strategies.

## Background

Due to the accumulation of various genetic mutations, cancer cells generally express various neoantigens,^1 2^ which are released into the tumor microenvironment (TME) following the death of the cancer cells and subsequently initiate the cancer-immunity cycle.^3^ Ideally, this cycle should result in the generation of abundant tumor-killing lymphocytes, thereby causing the regression of the tumor mass. Unfortunately, the cancer-immunity cycle does not perform optimally in most patients,^3^ since tumor cells often suppress antitumor immunity by activating a series of negative regulatory pathways.^4^ This process is known as cancer immunoediting, which includes three phases termed elimination, equilibrium and escape.^5 6^ Throughout cancer immunoediting, immunosuppressive mechanisms that enable cancer progression are acquired. Among these, programmed cell death 1 (PD-1) signaling is one of the most attractive targets as evidenced by the significant success of PD-1-based immunotherapy in cancer treatment.^7 8^

**Figure 1:**
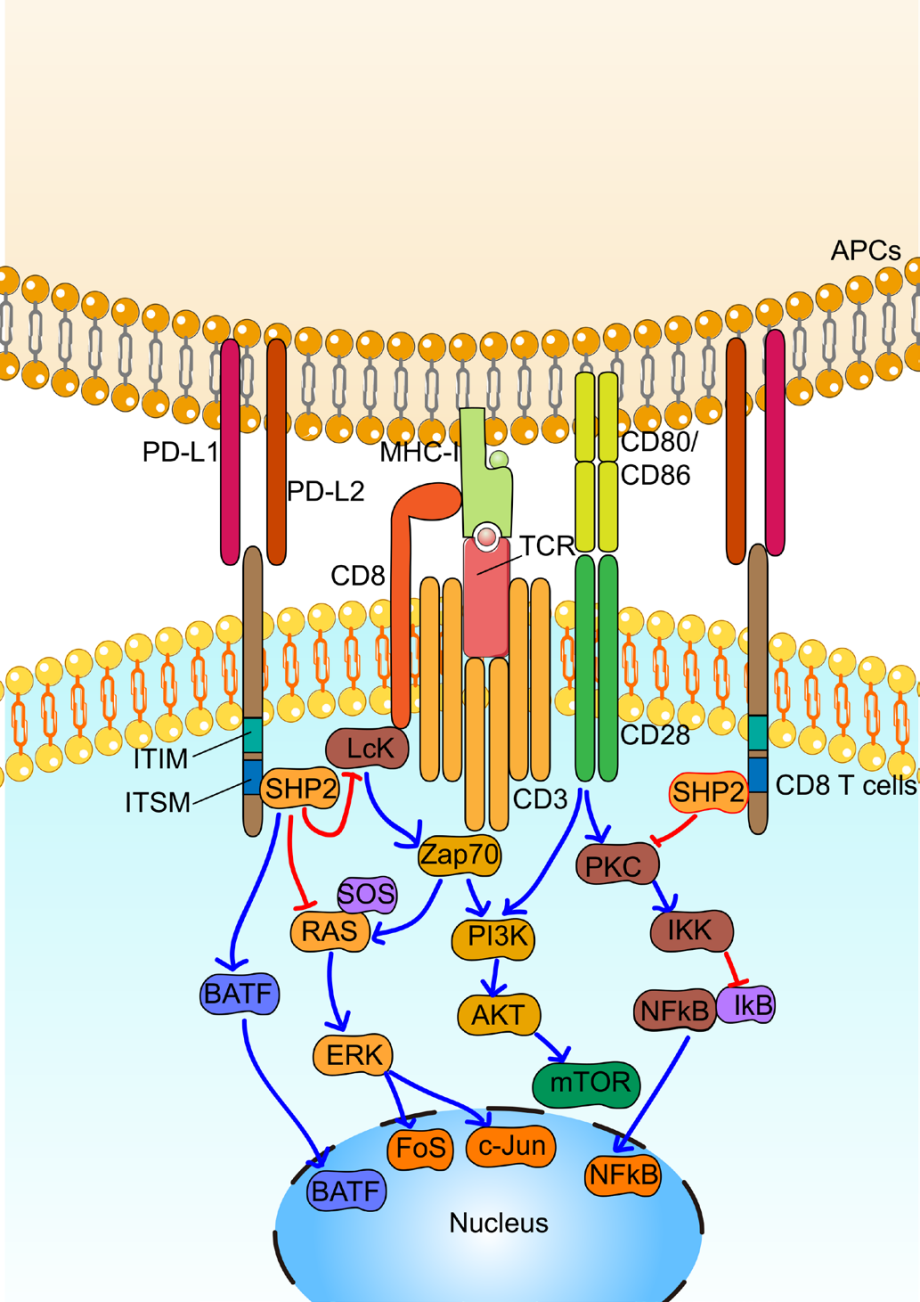
Canonical programmed cell death 1 (PD-1) signaling in CD8 T cells. By engagement with its ligands, including PD-L1 or PD-L2, PD-1 is phosphorylated at immunoreceptor tyrosine-based switch motif (ITSM) tyrosine residue sites, which leads to the binding of SHP2. Recruited SHP2 directly downregulate T cell receptor (TCR) signaling via dephosphorylation of proximal signaling elements, including PI3K, RAS and PKC, leading to decreased activation, proliferation, cytokine production and survival of CD8^+^ T cells. In addition, PD-1 signaling increases the expression of basic leucine zipper transcriptional factor ATF-like factor (BATF), which affects differentiation of immune cells. APC, antigen-presenting cell; ITIM, immunoreceptor tyrosine-based inhibitory motif.

PD-1 receptor was first cloned by Ishida *et al* in 1992.^9^ It is primarily expressed on T cells on activation. Two tyrosine motifs are present in the cytoplasmic domain of PD-1, including immunoreceptor tyrosine-based switch motif and immunoreceptor tyrosine-based inhibitory motif. Binding of PD-L1 and PD-L2 ligands to PD-1 induces phosphorylation of PD-1 at the tyrosine residues, leading to its interaction with SHP2.^10^ Historically, it was generally accepted that the recruited SHP2 downregulates T cell receptor (TCR) signaling via the dephosphorylation of downstream signaling regulators, which in turn suppresses the activation, proliferation, cytokine production and survival of T cells.^11^ However, recent studies suggest PD-1-SHP2 suppresses that T cell function primarily by favoring dephosphorylation of CD28 signaling over dephosphorylation of TCR signaling.^12 13^ PD-L1 is broadly expressed in somatic cells, while PD-L2 is primarily expressed by antigen-presenting cells (APCs). Driven by hypoxia and inflammatory cytokines, PD-L1 is overexpressed in the TME, along with an elevated expression of PD-1 on tumor-infiltrating lymphocytes, resulting in the disruption of the cancer-immunity cycle.^14^ Due to the well-established role of PD-1 on tumor-infiltrating cytotoxic T cells and conventional CD4 T cells, we designated this pathway, canonical PD-1 signaling (figure 1). In fact, PD-1 blockade therapy has been developed based on the well-established knowledge regarding canonical PD-1 signaling, and has achieved great success in treating different cancers.^15–17^

However, canonical PD-1 signaling is not the only type that exists in TME. For instance, in tumors containing tumor-infiltrating lymphocytes expressing heterogenous PD-1^18^ and exhibiting frequent loss of human leukocyte antigen-Ⅰ (HLA-I) expression,^19^ such as Hodgkin’s lymphoma, PD-1 blockade therapy remains highly responsive.^18^ Meanwhile, a small fraction of patients with cancer exhibits rapid cancer progression during PD-1 blockade therapy, also known as hyperprogressive disease (HPD).^20^ Furthermore, increasing evidence indicates that PD-1 is not only expressed by CD8 or CD4 conventional T cells, but also by other cell types, including tumor cells (table 1),^21 22^ as well as many types of stromal cells (table 2), consisting of regulatory T cells (Tregs),^23 24^ B cells,^25^ macrophages,^26^ natural killer (NK) cells^27^ and dendritic cells (DCs),^28 29^ indicating the probable influence of PD-1 blockade therapy on these diverse cell types. Based on the current knowledge, PD-1 signaling in these cell types is distinct from canonical PD-1 signaling, both in terms of function and associated molecular pathways; hence, we termed the PD-1 signaling occurring in these alternate cell types as non-canonical PD-1 signaling. This review focuses on the recent advances on non-canonical PD-1 signaling and aims to broaden the knowledge in the field of oncoimmunology.

**Table 1:** Non-canonical programmed cell death 1 (PD-1) signaling in cancer cells

Cancer type	Biology effect	Potential implication in clinic	Ref
Human melanoma (tumor tissue, cell lines), mouse melanoma (cell lines)	Promoting tumorigenesis by activating mTOR signaling	Contribution of melanoma PD-1 to the efficacy of PD-1 blockade therapy	22
Human hepatoma (tumor tissue, cell lines), mouse hepatoma (cell lines)	Promoting tumorigenesis by activating mTOR signaling	Contribution of hepatoma PD-1 to the efficacy of PD-1 blockade therapy	131
Human pancreatic cancer (tumor tissue, cell lines)	Promoting proliferation of cancer cells by targeting CYR61/CTGF via hippo pathway	Pancreatic cancer cell-intrinsic PD-1 correlate with poor prognosis	132
Human NSCLC (tumor tissue, cell lines), mouse NSCLC (cell line, M109)	Inhibiting proliferation of NSCLC cells	Contribution of NSCLC-intrinsic PD-1 signaling to HPD during PD-1 blockade therapy	133
Human lung cancer (tumor tissue, cell lines), human CRC (cell lines)	Inhibiting proliferation of cancer cells by suppressing AKT and ERK signaling (lung cancer cells and CRC cells)	Contribution of cancer cell-intrinsic PD-1 signaling to HPD during PD-1 blockade therapy	21

CRC, colorectal cancer; HPD, hyperprogressive disease; NSCLC, non-small cell lung cancer.

**Table 2:** Non-canonical programmed cell death 1 (PD-1) signaling in stromal cells

Cell type	Cancer type	Biology effect	Potential implication in clinic	Ref
Tregs	Human gastric cancer, mouse implanted tumor model (B16F0)	Promoting Tregs proliferation and immunosuppressive activity	Contribution of PD-1+ Tregs to HPD during PD-1 blockade therapy	23
	Mouse implanted tumor model (B16F10)	PD-1 signaling maintain the expression of FOXP3 through proteolytic pathway	NA	45
B cells	Human hepatoma, mouse orthotopic hepatoma (Hepa1-6)	Promoting tumor growth via secretion of IL-10	Contribution of B cells to efficacy of PD-1 blockade therapy.	25
NKs	Mouse implanted tumor model (RMA-S, CT26, 4T1)	Suppressing NKs mediated tumor control	Contribution of NK cells to efficacy of PD-1 blockade therapy.	27
	Human head and neck cancer	Inhibiting activation and cytotoxicity of NKs	Contribution of NK cells to efficacy of PD-1 blockade therapy.	144
TAMs	Human colorectal cancer and mouse implanted tumor model (CT26)	Inhibiting phagocytic capacity against tumor cells	Contribution of TAMs to efficacy of PD-1 blockade therapy.	26
	Human gastric cancer	Inhibiting phagocytic capacity against tumor cells	PD-1+ TAMs infiltration correlate with unfavorable prognosis in gastric cancer	118
Myeloid cells	Mouse implanted tumor model (B16F10, MC38)	Inhibiting differentiation of myeloid cells by restraining cholesterol.	Contribution of myeloid cells to efficacy of PD-1 blockade therapy.	119
DCs	Human ovarian cancer (tumor tissue and ascites), mouse implanted tumor model (ID8, intraperitoneally)	Inhibiting NF-kB-mediated antigen presentation in a SHP-2-independent manner	Contribution of NKs to efficacy of PD-1 blockade therapy.	28
	Human ovarian cancer	Promoting IL-10 production	Combined PD-1 blockade with IL-10 neutralization shows synergistic effect.	29
	Mouse implanted tumor model (ID8, intraperitoneally)	Promoting polarization toward an immunosuppressive and immature state by inhibiting NF-kB.	NA	124

DCs, dendritic cells; HPD, hyperprogressive disease; IL, interleukin; NA, not available; NKs, natural killer cells; TAMs, tumor-associated macrophage; Treg, regulatory T cells.

### Roles of PD-1 signaling in distinct cell types

#### Regulatory T cells

FOXP3-expressing Tregs are essential for the maintenance of peripheral tolerance; however, their suppressive effect can help tumor cells evade host immunity.^30 31^ A growing body of evidence suggests that Tregs are frequently accumulated in various tumor tissues by chemotaxis^32^ or driver gene alteration.^33 34^ Their intensity of infiltration is correlated with poor disease prognosis.^35 36^ Further, the infiltration of Tregs into inflamed tumors along with massive infiltration of CD4 effector T cells and cytotoxic T lymphocytes, has been reported in melanoma.^37 38^ In such cases, Treg infiltration correlates with a favorable outcome in patients with cancer.^39^ Furthermore, tumor-infiltrating Tregs are highly heterogeneous in terms of their functional state and stability, which might contribute to their variable correlation with prognosis of patient with cancer.^40 41^ Although further studies are needed to explore these phenomena, current reports demonstrate that targeting Tregs could be useful for the treatment of cancers.^42^ However, provided their potent role in the maintenance of peripheral tolerance, it is important to specifically target tumorous Tregs, while leaving the Tregs in other peripheral compartments unaffected, or minimally affected, to minimize the risk of autoimmune disorders.

In addition to conventional CD4 and CD8 T cells, a subset of Tregs also expresses high levels of PD-1.^23 43–48^ PD-1 on conventional CD4 T cells inhibits TCR signaling, which is essential for the survival and maintenance of the suppressive activity of Tregs,^49^ indicating that PD-1 expression on Tregs may inhibit their activation and suppressive activity. This hypothesis has been validated in a mouse model of autoimmune pancreatitis, which demonstrated that PD-1-deficient Tregs exhibited enhanced immunosuppressive activity compared with PD-1-sufficient Tregs.^50^ Furthermore, systemic PD-1 deficiency in mice was shown to cause autoimmunity,^11^ whereas, conditional PD-1 knockout on Tregs, particularly in the T follicular regulatory subset, resulted in their proliferation and enhanced immunosuppressive activity.^51^ Moreover, a preclinical study demonstrated that LKB1-deficient Tregs overexpress PD-1, as detected by flow cytometry (antibody clone, J43), while PD-1 blockade promotes the suppressive activity of Tregs, which in turn inhibits T helper 2-mediated immune responses.^24^ Additionally, a study by Takahiro *et al* demonstrated that during treatment with anti-PD-1 mAb, 4 of the 36 patients with gastric cancer succumbed to HPD.^23^ The patients with HPD had a massive infiltration of proliferating activated effector Tregs (eTregs), while patients without HPD had comparatively lower eTreg accumulation. Further, tumorous eTregs exhibited high expression of PD-1, as detected by flow cytometry (antibody clone: MIH4). Using human samples, the authors demonstrated that treatment with anti-PD-1 antibody increased the proliferation and immunosuppressive activity of Tregs in vitro.^23^ Furthermore, genetic ablation of PD-1 in murine Tregs increases their suppressive activity against antitumor immunity in vivo. Recently, the same group reported that PD-1 blockade reactivates CD28 and TCR signals both in CD8 T cells and Tregs. Intriguingly, they demonstrated that PD-1 expression balance of CD8 T cells and Tregs could predict response to PD-1 blockade therapy.^52^

**Figure 2:**
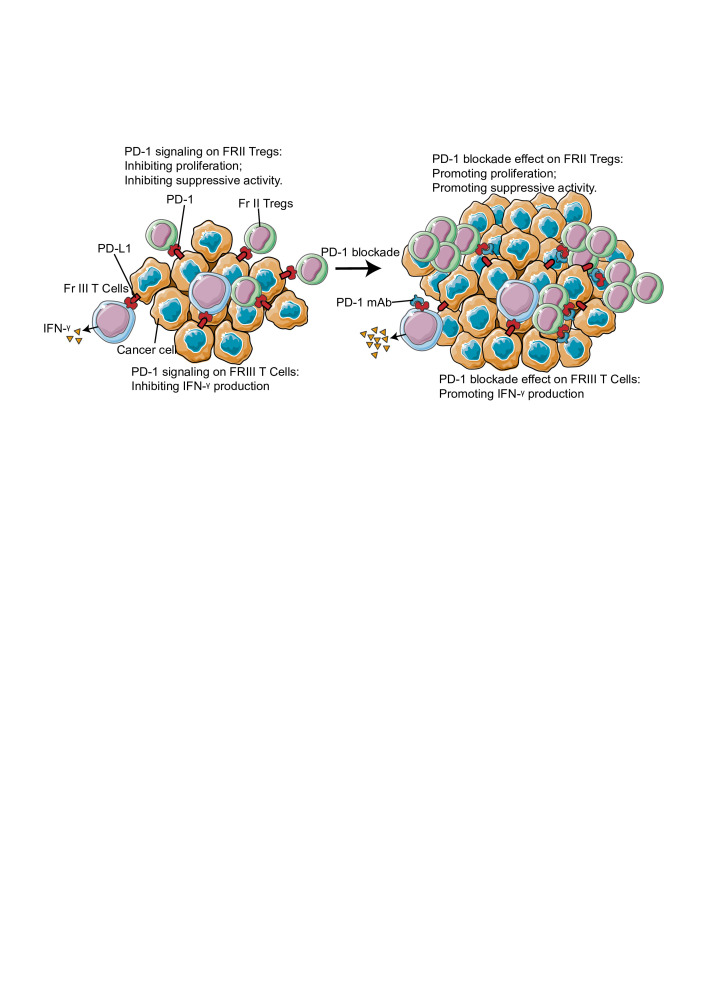
Role of non-canonical programmed cell death 1 (PD-1) signaling in tumorous Treg. PD-1 signaling inhibit proliferation and suppressive activity of tumorous Fr-II Tregs, while inhibiting IFN-γ production of tumorous Fr-III Tregs. In tumor, the major Treg population is Fr-II Treg. Thus, blockade of PD-1 signaling boost Fr-II Treg by promoting its proliferation and suppressive activity and potentially leads to hyperprogressive disease (HPD). IFN, interferon.

The data discussed thus far has suggested that PD-1 suppresses the proliferative and immunosuppressive properties of Tregs. However, opposite effects have also been observed for PD-1 expression on Tregs.^43 46^ For instance, in the case of tumors and chronic viral infections, PD-1 is essential for maintaining FOXP3 expression on Tregs through a proteolytic pathway.^45^ As FOXP3 is critical for the maintenance of the suppressive function of Tregs, PD-1 on Tregs is believed to maintain its immunosuppressive functions. Furthermore, using a chronic graft versus-host disease (cGVHD) model, Takeru *et al* demonstrated that a low dose of interleukin (IL)-2 induces PD-1 expression on Tregs, particularly in the Irving L. Weissman 44^+^CD62L^+^ central-memory subset. Moreover, PD-1-deficient Tregs exhibit rapid proliferation following IL-2 administration, however, eventually become proapoptotic.^43^ In glioblastoma, PD-1^high^ Tregs have been identified as a population of dysfunctional and exhausted Tregs, secreting interferon (IFN)-γ.^47^ Interestingly, administration of anti-PD-1 antibodies further enhanced the secretion of IFN-γ from Tregs. The contradictory roles of PD-1 on peripheral Tregs indicate that the effects elicited by PD-1 signaling on Tregs are context dependent. For instance, in a cGVHD model, stimulation with IL-2 at a low dose activated PD-1 signaling, while blockade of PD-1 signaling promoted apoptosis of Tregs, suggesting a potential role for PD-1 signaling in promoting, rather than suppressing, the immunosuppressive activity of Tregs in the cGVHD model. Nevertheless, the apoptotic Tregs in tumors exhibit superior immunosuppressive activity,^53^ indicating that PD-1 signaling blockade might enhance their immunosuppressive activity within the TME. Another possible reason for the differential behavior of Tregs is their heterogeneity. Although, that FOXP3 is highly specific to Tregs,^54^ studies suggest that FOXP3^+^CD4^+^ T cells are functionally and phenotypically heterogeneous, and consist of suppressive and non-suppressive T cells.^35 42 55 56^ For example, FOXP3^+^CD4^+^ T cells in colorectal cancer can be classified into three subsets based on their expression levels of CD45RA and FOXP3: Fraction I (Fr-I, FOXP3^low^CD45RA^+^), Fraction II (Fr-II, FOXP3^high^CD45RA^-^), and Fraction III (Fr-III, FOXP3^low^CD45RA^−^). Fr-I cells, representing naïve Tregs, differentiate into highly suppressive and functionally stable effector Tregs or Fr-II cells in response to stimulation by tumor antigen. In contrast, Fr-III cells are not suppressive and secrete inflammatory cytokines.^35^ Therefore, PD-1 signaling may simultaneously inhibit inflammatory cytokine production by Fr-III cells and impair the immunosuppressive activity and proliferation of Fr-II Tregs. These data highlight a need to accurately explicate the differential roles of PD-1 on various Treg subsets under distinct microenvironments. The clinical benefit of PD-1 blockade therapy needs to be assessed accordingly (figure 2).

### B cells

B cells are abundantly present in the TME.^57 58^ A recently conducted single-cell RNA-sequencing study on human lung cancer stromal cells demonstrated that B cells were the most enriched cell type in tumor stroma.^59^ However, the precise role of B cells in tumor immunity is debatable, as paradoxical roles have been reported. On one hand, tumor-infiltrating B cells have been reported to promote tumor growth by producing inhibitory cytokines, including IL-10 and TGF-β^60 61^ and by interacting with either immune cells via PD-L1^60^ or tumor cells through CD40/CD154 signaling, as shown in hepatocellular carcinoma (HCC).^62^ On the other hand, tumor-infiltrating B cells reportedly delay tumor growth by secreting antibodies against the tumor,^63^ producing proinflammatory cytokines, including IL-12,^64^ antigen presentation,^65^ or inducible T cell costimulator ligand (ICOSL) / inducible T cell costimulator (ICOS interaction.^66^ The contrasting roles of tumor-infiltrating B cells may be caused by their high heterogeneity.^59^ Interestingly, recent studies suggest the involvement of B cells in PD-1 blockade therapy response,^67–69^ showing clonal expansion, and a unique functional state of B cells as responders to PD-1 blockade therapy.^68^ Although the underlying mechanism remains largely unknown, these studies suggest a potential role of PD-1 signaling in controlling the functions of tumor-infiltrating B cells.

**Figure 3:**
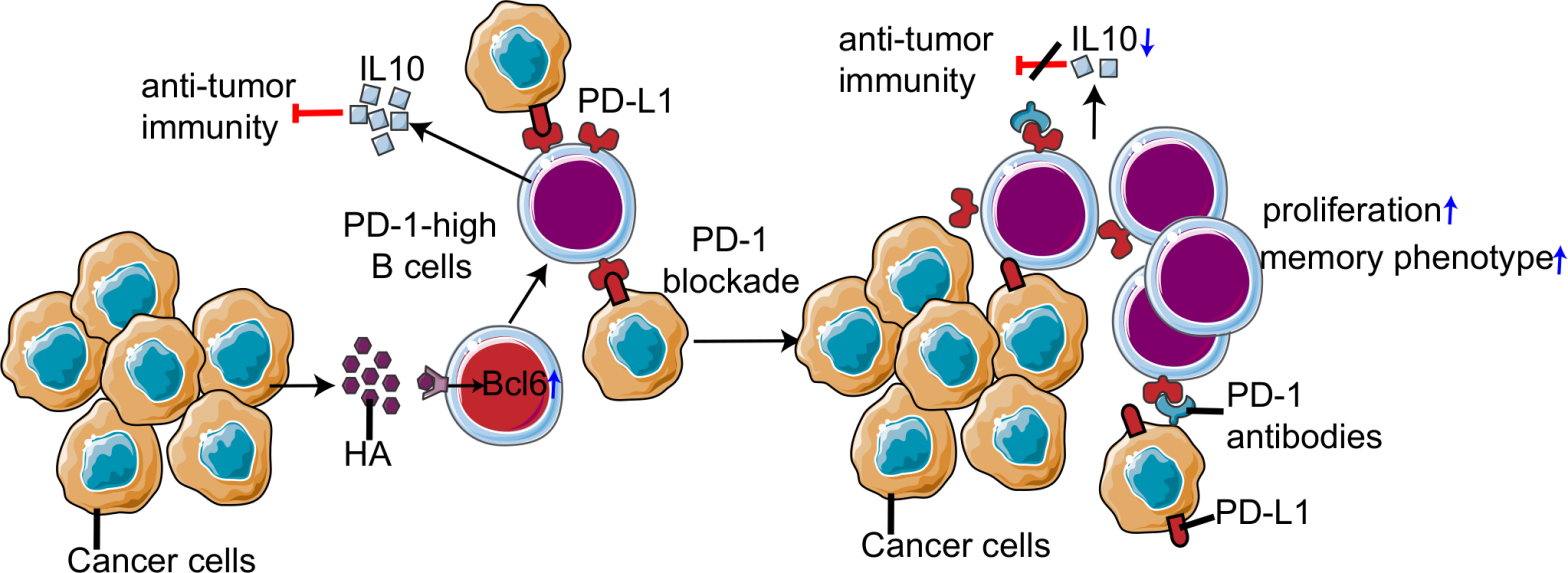
Non-canonical programmed cell death 1 (PD-1) signaling in tumorous B cells. Hepatocellular carcinoma (HCC) factors induce PD-1^high^ B cells through TLR4-driven Bcl-6 upregulation. By engagement with PD-L1, non-canonical PD-1 signaling in B cells inhibits antitumor immunity by enhancing IL-10 production. PD-1 blockade decreases interleukin (IL)-10 production and promotes proliferation and differentiation into a memory phenotype of B cells, which may influence efficacy of PD-1 blockade therapy.

In addition to T cells, B cells have also been reported to express PD-1.^70 71^ The role of PD-1 on B cells was first observed in PD-1^−/−^ mice, which exhibited moderate splenomegaly and increased B cell proliferation in response to anti-IgM antibody stimulation.^72^ Further, resting human B cells express PD-1 at a basal level, while its expression is rapidly induced on stimulation of the toll-like receptor 9 (TLR9) pathway by CpG-B.^70^ Subsequent PD-1/PD-L1 interaction inhibits the B cell receptor signaling pathway, resulting in the attenuation of cytokine production and proliferation of B cells.^71^ Recently, a novel tumor-promoting subset of B cells has been identified that expresses high levels of PD-1 in human HCC.^25^ These PD-1^high^ B cells constitute approximately 10% of the total B cell population in advanced HCC. Unlike conventional regulatory B cells (Bregs), PD-1^high^ B cells exhibit a CD5^high^CD24^−/−^CD27^high/+^CD38^dim^ phenotype. PD-1 expression is highly induced by hyaluronan fragments and TLR agonists, including Pam3CysSK4, lipopolysaccharide (LPS), and oligodeoxynucleotides containing CpG motifs.^25^ TLR4-induced BCL6 upregulation plays a dominant role in the induction of PD-1 on B cells. The interaction between PD-L1 and PD-1^high^ B cells induces the expression of IL-10, a well-defined immune-suppressive cytokine. These data suggest that the contribution of B cells in PD-1-based immunotherapy should be critically evaluated and PD-1 signaling must be further investigated in different B cell subsets (figure 3). Besides, follicular T-helper cell (Tfh), which exhibit high PD-1 expression, plays a critical role in promoting maturation of B cells.^73^ Given PD-1 signaling is essential for positioning and function of Tfh, it is reasonable to speculate that PD-1 blockade therapy could affect B cells in an indirect manner.^74^ In this regard, the role of Tfh on B cells in PD-1 needs blockade therapy which need further study.

### NK cells

NK cells were first identified in 1975.^75 76^ Since that time, they have been classified as lymphocytes based on their morphology, phenotype, and origin, and are considered as a part of innate immunity due to the absence of antigen-specific receptors.^77^ The functional status of NK cells is mediated by the cumulative effect of multiple activating and inhibitory receptors. When activated, NK cells kill virus-infected cells and malignant tumor cells in an major histocompatibility complex (MHC) non-restricted manner.^78^ NK cells also secrete various inflammatory cytokines with antitumor effects, including IFN-γ and tumor necrosis factor (TNF)-α. Indeed, the presence of NK cells in solid tumors has been described as a good prognostic factor.^79–82^

Studies have suggested three potential contributions made by PD-1 signaling in NK cells during PD-1 blockade therapy: (1) HLA-I is generally not expressed in human cancer cells, resulting in no interaction between HLA-I and CD8 T cells^83 84^; however, PD-1-based immunotherapy is effective in these tumors. For instance, 79% (85/108) of the classical Hodgkin’s lymphomas (cHL) exhibit low to no expression of MHC class I molecules.^19^ Meanwhile, PD-1 blockade therapy is highly effective in the treatment of relapsed or refractory cHL.^18^ (2) Tumor cells with high tumor mutation burden (TMB) are more likely to be recognized by CD8 T cells, and patients carrying tumors with TMB, such as malignant melanoma^85^ and non-small cell lung cancer,^86^ are more likely to benefit from PD-1 blockade therapy.^2^ However, even in tumors with low TMB, including malignant melanoma and non-small cell lung cancer, a small fraction of patients are responsive to PD-1 blockade therapy.^85 86^ (3) Human NK cells express PD-1 in various cancers, including Hodgkin’s lymphoma.^87–89^ Hsu *et al*^27^ used different mouse models to investigate PD-1 signaling in NK cells and its role in PD-1-based immunotherapy. Interestingly, PD-1 was found to be expressed by tumor-infiltrating NK cells with an activated but exhausted phenotype. Furthermore, NK cells contributed to the antitumor effect of PD-1-based immunotherapy.^27^ However, this study had several limitations. For instance, although PD-1 expression on human NK cells has been well characterized, its expression on mouse NK cells remains debatable, as they do not express PD-1 even under robust activation conditions, such as cytomegalovirus infection.^90^ This study concluded the PD-1 expression on NK cells by flow cytometry, while a recent study suggested that dying immune cells express a nuclear antigen, which cross-reacts with mouse anti-PD-1 monoclonal antibody, leading to a false positive PD-1 staining on NK cells.^91^ Second, the molecular mechanisms underlying the effect of PD-1-based immunotherapy on NK cells remains unclear. Conversely, a recent study challenged the expression of PD-1 on mouse and human NK cells under diverse conditions.^92^ Overall, the expression and effect of PD-1 on NK cells remains unclear. However, combination therapies including PD-1 blockade and NK cell activation strategies are currently in clinical trials.^93^ Further studies are urgently needed to address the expression of PD-1 as well as its precise role in NK cells.

Lastly, considering that NK cells are a small minority of cells in the TME of multiple cancer types, other cell types, such as CD4 T cells^94 95^ and macrophages^96^ may function cooperatively with NK cells to exert antitumor effects during PD-1 blockade therapy. In a study of patients with relapsed or refractory cHL, the authors demonstrated that PD-L1 expression and MHC class II positivity on Hodgkin Reed-Sternberg cells are favorable prognostic biomarkers of PD-1 blockade therapy, which potentiate an alternative CD4 T cell-mediated mechanism of response to PD-1 blockade therapy.^97^ Accordingly, a study suggest that cytotoxic CD4 T cells are essential to the efficacy of PD-1 blockade therapy on MHC class II-expressing tumors.^98^ Intriguingly, a recent study suggested a tumor-promoting role for PD-L1 reverse signaling in promoting tumor cell growth, proliferation and metabolism in cHL, which may also participate in PD-1 blockade therapy.^99^

### Macrophages

Macrophages that infiltrate solid tumors are referred to as tumor-associated macrophages (TAMs).^100^ High expression of TAM markers, especially M2 markers, is generally associated with poor prognosis of patients with cancer.^100^ In most cases, bone marrow monocytes differentiate into TAMs following stimulation by TME factors, including cytokines and hypoxia.^101^ However, TAMs are also reportedly derived from myeloid progenitors present in the yolk sac.^102 103^ Additionally, monocyte-derived macrophages can be polarized following stimulation by cytokines and other environmental factors.

Macrophages are categorized as M1 or M2 based on their polarization. M1 refers to a proinflammatory state, which is generally driven by IFN-γ/LPS; while M2 refers to an anti-inflammatory state, generally mediated by IL-4 or IL-13.^100 104^ However, the M1/M2 model is an oversimplification and cannot precisely describe the polarized state of TAMs.^105^ In fact, TAMs express both M1 and M2 related markers concurrently.^26 106^ Therefore, provided the complexity of their origin and polarization state, TAMs exhibit high heterogeneity.^107^ Generally speaking, TAMs play a dominant role in promoting cancer progression by modulating nearly every aspect of tumor biology, including angiogenesis,^108^ metastasis,^109^ proliferation,^110^ immune suppression,^111 112^ inflammation^113^ and stem cell maintenance.^114^

Macrophages have been recently described as expressing PD-1 under specific conditions, such as tuberculosis,^115^ sepsis^116^ and zymosan-induced inflammation.^117^ However, its expression in macrophages is induced by TLR signaling, whereas in T cells, it is driven primarily by TCR signaling.^117^ PD-1 signaling in macrophages inhibits M1 polarization in-vitro via attenuation of STAT1/NF-κB phosphorylation.^117^ Furthermore, PD-1^−/−^ mice are markedly protected from lethal sepsis in vivo; however, the bactericidal effect is reversed, when macrophages are depleted by clodronate liposomes.^116^ Moreover, PD-1 blockade augments phagocytosis and intracellular killing activity of macrophages against BCG.^115^ Although these results reveal a potential inhibitory effect for PD-1 signaling on macrophages, the specific role of macrophage-associated PD-1 in tumor immunity remains unclear. In 2017, the research group of Sydney R. Gordon was the first to evaluate the expression and function of PD-1 on TAMs.^26^ Using flow cytometry and immunofluorescence, they reported that both human and murine TAMs express PD-1. Specifically, PD-1^+^ TAMs exhibited M2-like phenotype and accumulated in TME over time. Furthermore, in vitro and in vivo studies showed that PD-1^+^ TAMs had lower phagocytic properties, which were abrogated on PD-L1-knockout in mice. These results suggest that PD-1 is a functionally important M2-like marker. In fact, PD-1^+^ TAM infiltration correlates with poor prognosis in patients with human gastric cancer.^118^ Furthermore, PD-1 expression has been reported in murine tumorous myeloid cells using flow cytometry (antibody clone: RMP1-30). Intriguingly, PD-1 deletion in myeloid cells (PD-1^f/fLysMcre^ mice) effectively delays tumor growth, similar to that observed during global deletion of PD-1, whereas deletion of PD-1 in T cells (PD-1^f/fCD4cre^ mice) is less effective.^119^ These results indicate a crucial role for PD-1 in inhibiting the antitumor immunity of myeloid cells. Similarly, PD-1 expression was also reported in granulocyte/macrophage progenitors (GMPs), which increases during emergency hematopoiesis facilitating differentiation into myeloid-derived suppressor cells (MDSCs). Meanwhile, PD-1 deletion in myeloid cells (PD-1^f/fLysMcre^ mice) inhibits the accumulation of GMPs and MDSCs.^119^ Further, activation of ERK1/2, as well as the mTOR1 kinase complex by granulocyte colony-stimulating factor in myeloid cells, is inhibited by PD-1 expression. mTOR participates in the regulation of myeloid progenitor cell differentiation, while ERK1/2 controls the differentiation of APCs.^119^ Taken together, these data indicate that the effect of PD-1 blockade on TAMs should not be neglected, although it remains unclear whether patients with cancer with high PD-1^+^ TAM infiltration would also benefit from PD-1 blockade therapy.

### Dendritic cells

In spite of their paucity, DCs play a central role in the initiation of antigen-specific immunity and tolerance.^120^ Similar to other tumor-infiltrating stromal cells, DCs exhibit high heterogeneity.^120^ The functional specificity of DC subpopulations results from the expression of different receptors, including PD-1.^28 29 121 122^ Furthermore, various inflammatory factors induce PD-1 expression on DCs, which then suppresses innate immunity against bacterial infections by inhibiting IL-12 and TNF-α,^122^ and promotes apoptosis of activated DCs.^123^ Since PD-1 expression on DCs is induced by inflammatory factors, it is possible that tumor-infiltrating DCs also express PD-1, driven by chronic inflammation, a hallmark of cancer. By using flow cytometry and immunofluorescence, James *et al* observed a similar phenomenon of PD-1 expression in tumor-infiltrating DCs using an implanted tumor model of ovarian cancer. In murine species, immature PD-1^+^ DCs exhibit a classical DC phenotype (CD11c^+^ CD11b^+^ CD8^-^) that is suppressive, and respond weakly to danger signals. PD-1 signaling in mice has also been reported to inhibit NF-κB, a crucial signaling molecule involved in the maturation and activation of DCs.^124^ Similarly, PD-1^+^ DCs were also observed in human ovarian cancer, as determined by flow cytometry.^28^ Similar to its role in murine species, PD-1 expression on human DCs suppresses NF-κB-dependent cytokine release in a SHP-2-dependent manner. Further, PD-1 expression on DCs is induced by IL-10, a well-established suppressive cytokine, while PD-1 blockade leads to increased IL-10 production by DCs. In this regard, combined PD-1 blockade therapy with IL-10 neutralization induced a synergistic antitumor effect.^29^ These data are in agreement with that of another study, which suggested that PD-1 signaling in monocytes of HIV-infected individuals inhibits IL-10 production.^125^ Meanwhile, a recent study employed flow cytometry (antibody clone: EH12.2H7) to demonstrate that human tumorous DCs express PD-1. Moreover, PD-1 on DCs neutralizes PD-L1 in cis to inhibit canonical PD-1 signaling in T cells.^126^ However, these findings were exclusively based on in vitro experiments and, thus, may not reflect actual tumor-infiltrating DCs. Therefore, in vivo studies are warranted to examine the *cis* effect of PD-1 on DCs to modulate cancer immunity.

### Tumor cells

**Figure 4:**
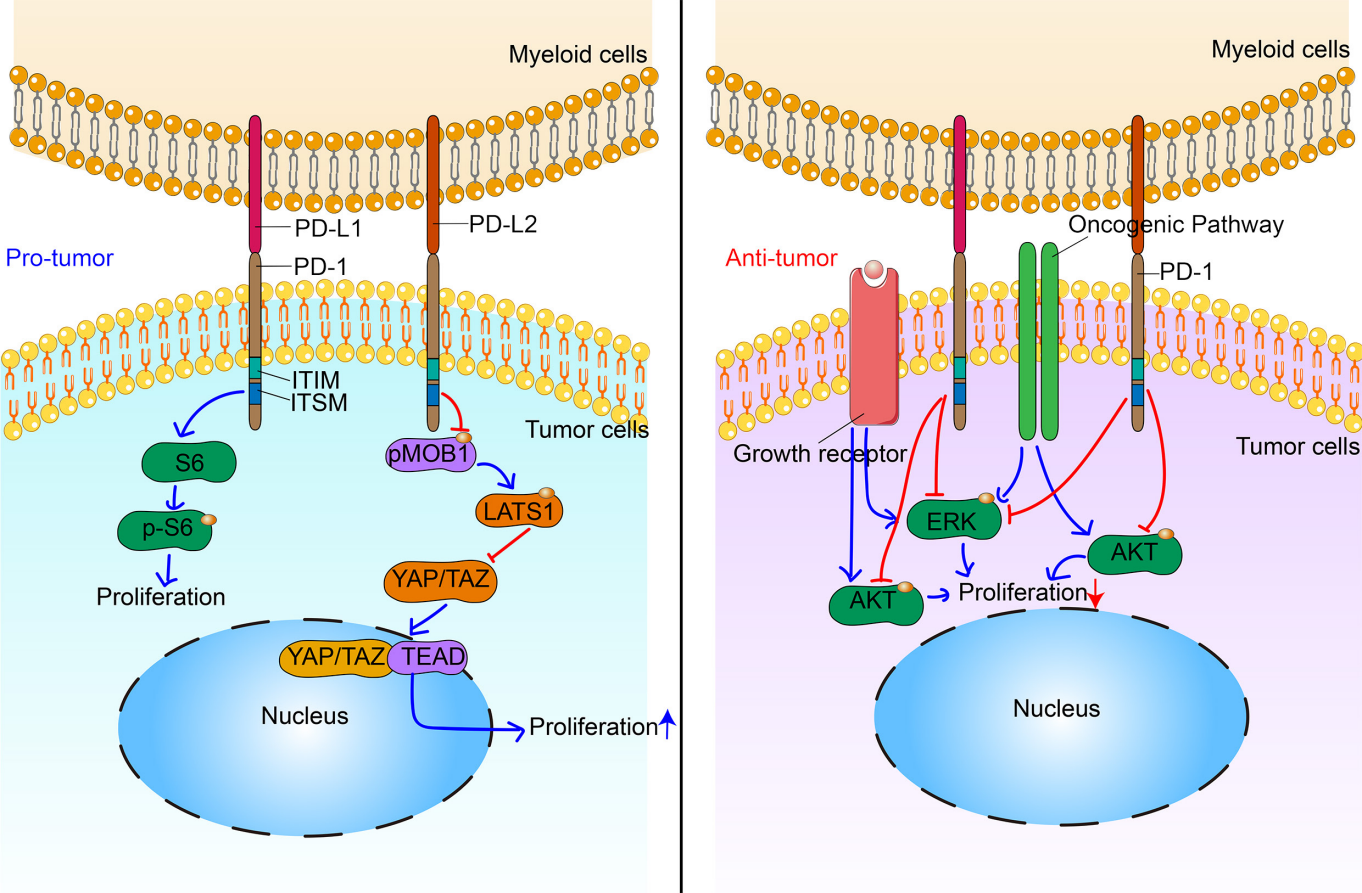
Multifaceted roles of non-canonical programmed cell death 1 (PD-1) signaling in tumor cells. In malignant melanoma, hepatocellular carcinoma and pancreatic cancer, non-canonical PD-1 signaling promotes proliferation of cancer cell via interacting with mTOR and Hippo signaling. In non-small cell lungcarcinoma (NSCLC), non-canonical PD-1 signaling inhibits proliferation of cancer cell via suppressing AKT and ERK signaling. ITIM, immunoreceptor tyrosine-based inhibitory motif; ITSM, immunoreceptor tyrosine-based switch motif.

Until 2010, the accepted dogma was that PD-1 is specifically expressed by cells of the hemopoietic lineage. However, by using flow cytometry and immunofluorescence, Tobias *et al* reported that a subpopulation of melanoma cells express PD-1, and that PD-1^+^ cancer cells are responsible for tumor initiation.^127^ In this study, PD-1 was primarily examined as a biomarker, enriched in ABCB5^+^ malignant melanoma-initiating cells. Meanwhile, the function of PD-1 signaling in melanoma cells remained largely unknown until 2015. Tobias *et al* further demonstrated that human and murine melanoma cells contain PD-1-expressing subpopulations by using flow cytometry, immunofluorescence, RT-PCR and western blotting.^22^ In melanoma cells, intrinsic PD-1 signaling plays a key role in tumor initiation in an immunosuppression-independent manner. By interacting with its cognate ligand, melanoma-PD-1 triggers the activation of downstream effectors (eg, ribosomal protein S6) of mTOR signaling, which further accelerate tumor growth. Interestingly, intrinsic PD-1 signaling in melanoma cells activates downstream mTOR signaling in a PI3K/AKT-independent manner, distinct from that observed in canonical PD-1 signaling. In T cells, interaction of PD-1 with PD-L1/PD-L2 inhibits TCR signaling via SHP-2 tyrosine phosphatase.^14^ SHP-2 expression is reported in a number of cancer cell types, including melanoma,^128^ breast cancer^129^ and glioblastoma.^130^ In cancer cells, SHP-2 may activate mTOR signaling^130^; hence, the effect of PD-1 signaling on mTOR activation might be tissue specific. Consistent with this, both murine and human HCCs were reported to express PD-1.^131^ HCC cell-PD-1 promotes phosphorylation of eukaryotic initiation factor 4E (eIF4E) and ribosomal protein S6 (S6), leading to enhanced tumor cell proliferation.^131^ In addition, intrinsic PD-1 signaling in pancreatic cancer cells promotes tumor proliferation by decreasing the phosphorylation of MOB1, a central component of the Hippo signaling pathway.^132^ These data demonstrate that tumor cell-intrinsic PD-1 signaling exerts a protumor effect by activating mTOR in melanoma, HCC and pancreatic cancer cells (figure 4).

On the contrary, tumor cell-intrinsic PD-1 has been reported as a tumor suppressor gene in non-small cell lung carcinoma (NSCLC) and colon cancer.^21 133^ A previous study reported a 61-year-old woman diagnosed with stage IV NSCLC who was unresponsive to several chemotherapies and experienced rapid disease progression after receiving radiotherapy combined with pembrolizumab.^133^ PD-1 and PD-L1 expression in the irradiated tumor tissue biopsies obtained prior to pembrolizumab treatment were assessed by immunohistochemistry (IHC). Unexpectedly, the cancer cells displayed diffuse PD-1 staining. Meanwhile, RNA-sequencing data from lung cancer cell lines validated PD-1 expression in only 7 of 236 cell lines considered. Further, treatment with PD-1 antibodies (clone RMP1-14, rat IgG2a) accelerated the growth of M109 murine NSCLC in vitro and in vivo. However, the underlying mechanisms associated with these phenomena remain unclear. In line with this, a recent study shows that tumor cell-intrinsic PD-1 expression suppresses the canonical signaling pathways, including AKT and ERK1/2 in NSCLCs and colon cancer, while mTOR signaling remains unaffected.^21^ Treatment with either nivolumab (anti-PD-1 antibody) or ateolizumab (anti-PD-L1 antibody) enhances the growth of NCI-H1299 transplanted tumors in NOD-SCID IL-2 receptor gamma null mouse, one of the most immunodeficient mouse strains (figure 4). However, further studies are needed to explore the underlying molecular mechanisms responsible for these contradictory effects mediated by tumor cell-intrinsic PD-1 in melanoma, HCC and NSCLCs.

**Figure 5:**
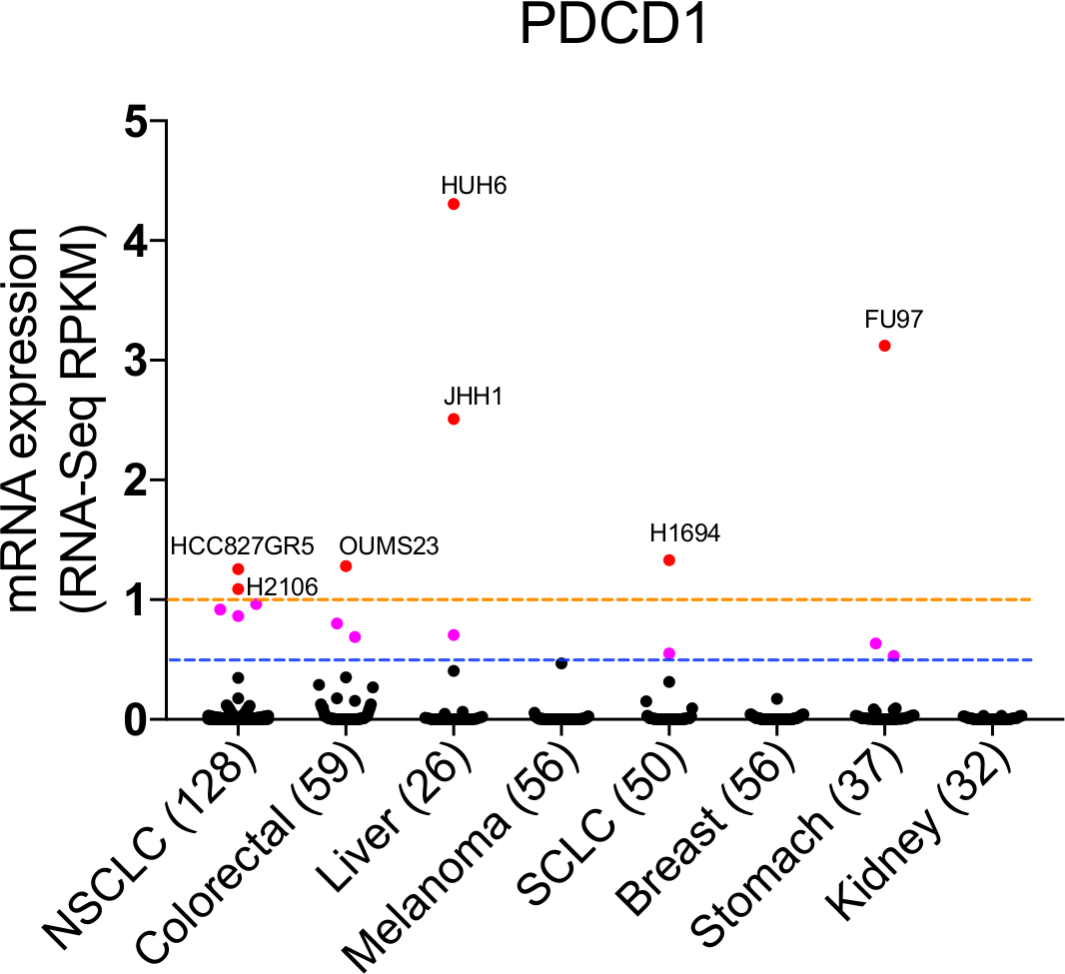
Expression of PDCD1 in cancer cell lines. PDCD1 mRNA expression (RNA-seq, RPKM) data of cancer cell lines (Cancer Cell Line Encyclopedia, Broad, 2019) were downloaded from cBioPortal (https://portals.broadinst-itute.org/ccle/) in 27 March 2020. PDCD1 expression of epithelium-transformed cancer was analyzed. Note: breast cancer cell line (DU4475, RPKM=48.02668) was not included in our panel as it would make this panel less informative.

Furthermore, a recent study reported the potential for obtaining false-positive PD-1 staining in melanoma cells, including B16F10^91^ due to cross reaction of antibodies with a nuclear antigen. These results raised the question regarding whether epithelial tumor cells truly express PD-1. Although evidence of PD-1 expression in tumor cells has been provided at the mRNA (qRT-PCR and RNA-sequencing), and protein (IHC, immunofluorescence, western blotting, and FACS) levels in previous studies,^21 22 127 131 133^ we re-evaluated the expression of *PDCD1* using the Cancer Cell Line Encyclopedia (Broad, 2019) in lung cancer (NSCLC and SCLC), colorectal, breast, melanoma, kidney, stomach and liver cancer cell lines. Our data suggest that majority of epithelium-derived cancer cells express *PDCD1* at extremely low, to undetectable, levels (RPKM <1; an RPKM of 1 is considered as a threshold and is equivalent to one mRNA copy per cell^134^) (figure 5). The low to undetectable expression of *PDCD1* in cancer cell lines may be attributable to its expression only in a subpopulation of cancer cells.^21 22^ Therefore, single-cell RNA-sequencing of cancer cells might be helpful in validating the expression of *PDCD1* in specific cancer cell subpopulations and characterizing the signaling involved in the regulation of *PDCD1* in cancer cells.

### Implications of non-canonical PD-1 signaling in cancer therapy

PD-1-based immunotherapy has achieved great clinical success and has been approved for the treatment of various cancers.^16 17 135 136^ However, only a fraction of the patient population benefits from PD-1 blockade therapy.^137^ Even with the help of validated biomarkers, including PD-L1 and TMB, the response rate remains low (eg, approximately 40% in patients with NSCLC with PD-L1 expression in at least 50% of the cancer cells^16^). Furthermore, a small fraction of patients with cancer receiving PD-1-based immunotherapy succumbs to HPD.^20^ Hence, there is a critical need to determine the cause for the beneficial or deleterious effects of PD-1 blockade therapy in patients.

Note, although treatment with anti-PD-1 antibodies affects all PD-1-expressing cells, since conventional CD4 T and CD8 T cells often account for the dominant cells expressing PD-1, effects of PD-1 blockade on other PD-1-expressing cells have been neglected. In fact, the antitumor effect induced by PD-1 blockade therapy is a cumulative effect of its influence on all PD-1-expressing cells. In this regard, cells associated with non-canonical PD-1 signaling should also be evaluated as biomarkers for combinatorial therapeutic strategies.

### Potential biomarkers for PD-1 blockade therapy

**Figure 6:**
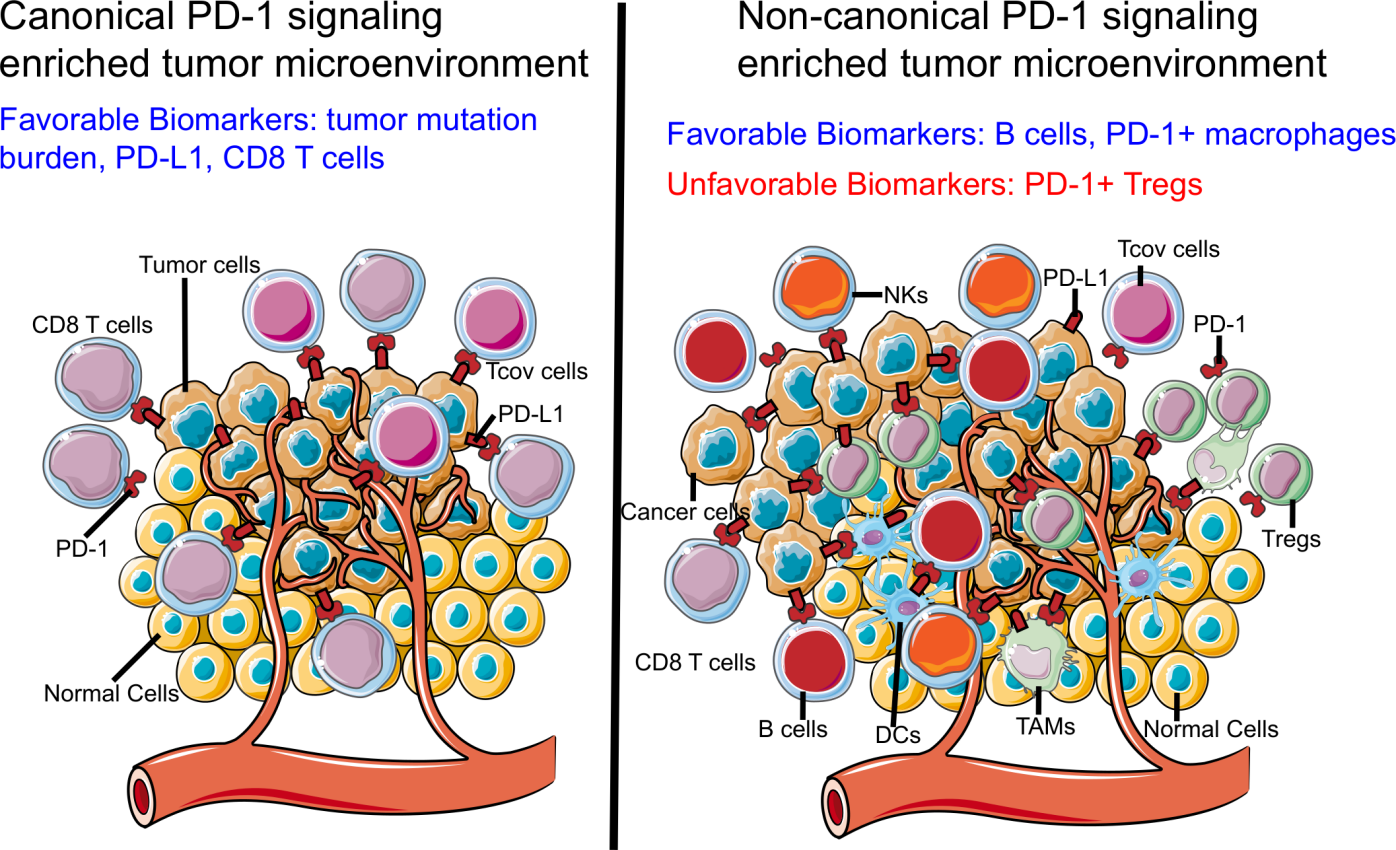
Tumor microenvironment (TME) characterized by canonical or non-canonical programmed cell death 1 (PD-1) signaling. Based on the different status of PD-1 signaling, we classified TME into two types: canonical PD-1 signaling-enriched TME (left) and non-canonical PD-1 signaling-enriched TME (right). Canonical PD-1 signaling-enriched TME is characterized by high infiltration of CD8 T cells and conventional CD4 T cells. Biomarkers for PD-1 blockade therapy including PD-L1 and TMB may work optimally in this type TME. However, biomarkers for non-canonical PD-1 signaling-enriched TME remains largely unknown. DC, dendritic cell; NK, natural killer; TAM, tumor-associated macrophages.

To date, most of the validated biomarkers related to PD-1 blockade therapy, including PD-L1,^138 139^ TMB^86 136^ and CD8 T cell infiltration,^140^ are based on the mechanisms underlying CD8 T cell-driven antitumor activity. However, even with the help of these biomarkers, it remains difficult to identify responders and non-responders of PD-1 blockade therapy.^11^ For example, PD-L1 expression does not always correlate with clinical outcomes. Although patients with advanced NSCLC with PD-L1^+^ tumors (PD-L1 expression on at least 50% of tumor cells) have been reported to respond well to PD-1 blockade therapy, only 44.8% of patients benefit from this therapy.^15^ Similar results have been reported for TMB as a biomarker for PD-1 blockade therapy, for which a group of patients with low TMB responded well to PD-1 blockade therapy.^85^ As mentioned earlier, since all PD-1-expressing cells are affected by PD-1 blockade therapy, it is helpful to consider the effect of PD-1 antibodies on these additional PD-1-expressing cells when developing novel biomarkers. For example, Hodgkin’s lymphoma lacks MHC-I expression and has low TMB, making it challenging to generate effective antitumor CD8 T cell responses. Nevertheless, it responds well to PD-1 immunotherapy. These results suggest that other PD-1-expressing cell types, such as B cells and TAMs, may serve as novel biomarkers for PD-1 blockade therapy (figure 6). Interestingly, a few recent studies reveal that B cells are the strongest prognostic factor in patients with melanoma,^67 68^ sarcoma^69^ and renal cell carcinoma^68^ receiving PD-1 blockade therapy, even though the tumors have low level of CD8 T cell infiltration.^69^ Moreover, patients having tumors with high Treg infiltration should avoid anti-PD-1 antibody monotherapy, as it may lead to HPD due to increased expansion and immunosuppressive activity of Tregs.^23^ Further, the effect of PD-1 blockade therapy in such patients can be determined using patient derived xenograft models. Lastly, given a recent study demonstrated that PD-1 expression balance of T cells could predict efficacy of PD-1 blockade therapy,^52^ it would be valuable to determine the association between PD-1 expression balance of all PD-1-expressing cell subsets and clinical response of patients receiving PD-1 blockade therapy.

### Combination therapy

As the response rate to PD-1 blockade monotherapy remains low, combination therapy has emerged as a recent trend in cancer treatment.^141^ In this context, targeting non-canonical PD-1 signaling may be considered as a novel strategy. For example, expansion of PD-1-expressing Tregs during PD-1 blockade therapy serves as a potential cause of HPD. Hence, targeting Tregs could be an important strategy for the prevention of HPD, while enhancing the efficacy of PD-1 blockade therapy. In fact, combination of nivolumab (anti-PD-1 antibody) and ipilimumab (anti-CTLA4 antibody) has resulted in higher response rates and longer progression-free survival in malignant melanoma patients than nivolumab or ipilimumab alone.^135^ Further, the HPD rate was also reduced in the combination group,^135^ which was likely due to the tumorous Treg-depleting effect of ipilimumab.^142^ In addition, a previous study reported that CCR4 is expressed specifically on the surface of tumorous effector Tregs, and an anti-CCR4 mAb (mogamulizumab) effectively depletes tumorous Tregs.^42^ Further, a recent phase I clinical trial reported an acceptable safety profile for mogamulizumab and nivolumab combination therapy.^143^

## Conclusions

Due to the well-established role of canonical PD-1 signaling in T cells, PD-1-based immunotherapy has achieved great clinical success. However, the response rate remains low, while a small group of patients succumb to HPD during PD-1 blockade therapy. Hence, canonical PD-1 signaling does not provide a complete explanation for the effect. Multiple studies have reported the expression and function of PD-1 on B cells, TAMs, DCs, NKs, Tregs and cancer cells, although the role of PD-1 in these cells remains unclear. Furthermore, it has been shown that non-canonical PD-1 signaling plays an important role in PD-1 blockade therapy response and HPD. Thus, a detailed understanding of non-canonical PD-1 signaling may provide novel biomarkers for identifying responders and non-responders to PD-1 blockade therapy. Additionally, it may inform the directed exploration of strategies for combinational therapy to markedly enhance the efficacy of PD-1-based immunotherapy.
